# Supplementary material for: Exact Calculation of the Joint Allele Frequency Spectrum for Isolation with Migration Models
Source: Genetics. 2017 Jul 10;207(1):241–53. doi: 10.1534/genetics.116.194019 (PMC5586375; doi:10.1534/genetics.116.194019)
Supplement: Supplementary file 1 [file 241FileS1.pdf]

Supplement to “Exact calculation of the joint allele frequency spectrum for isolation with migration models”

Andrew D. Kern[1] and Jody Hey[2]

June 30, 2017

[1]Department of Genetics, Rutgers University, Piscataway, NJ 08554, USA
[2]Department of Biology, Temple University, Philadephia, PA 19122 USA

pop1	pop2	lik	N_1	N_2	N_A	$4Nm_{12}$	$4Nm_{21}$	t_{div}	95% CI N_2	95% CI N_A	95% CI $4Nm_{12}$	95% CI $4Nm_{21}$	95% CI t_{div}
ED	GU	-104955.7618	63834.11	222269.94	80613.58	4.98	0.16	3939.55	13663.32	3703.80	0.14	0.13	255.89
ED	NG	-133735.8783	67163.37	207423.81	118095.48	4.33	0.79	4332.84	14956.00	5267.41	0.16	0.22	239.69
ED	SP	-116974.0971	56695.62	214161.24	88823.47	2.56	1.56	5577.46	15014.74	3896.12	0.10	0.17	254.84
ED	UG	-92419.45582	63696.49	162379.37	76674.47	4.91	0.73	3628.34	12351.57	3885.39	0.23	0.24	253.81
GU	NG	-186862.1182	312427.87	395230.05	150614.71	17.20	14.87	8496.82	94002.97	20344.21	2.14	6.87	1122.92
GU	SP	-178614.0678	334285.47	429910.17	120165.22	0.37	7.91	9985.27	21183.04	3644.12	0.39	0.58	358.13
GU	UG	-140765.3032	419695.00	348198.95	108844.22	12.17	9.93	7203.86	27632.06	5092.39	0.98	1.90	424.92
NG	SP	-215684.7144	295844.35	432935.59	166976.94	1.97	6.87	10582.02	20473.44	4889.41	0.25	0.43	430.50
NG	UG	-164929.6795	285763.09	339789.04	138162.32	12.92	2.60	8325.86	24895.30	6175.22	0.47	0.65	525.89
SP	UG	-151679.0982	396609.71	231793.18	111410.91	12.61	1.97	9257.11	11359.69	3173.21	0.33	0.49	375.11

Table S1: Parameter estimates from IM_CLAM for pairwise combinations of populations. Estimates of $4Nm$ are scaled in N_1 for each population comparison respectively. t_{div} is given in years, assuming 15 generations per year and and $u = 5.49 \times 10^{-9}$.

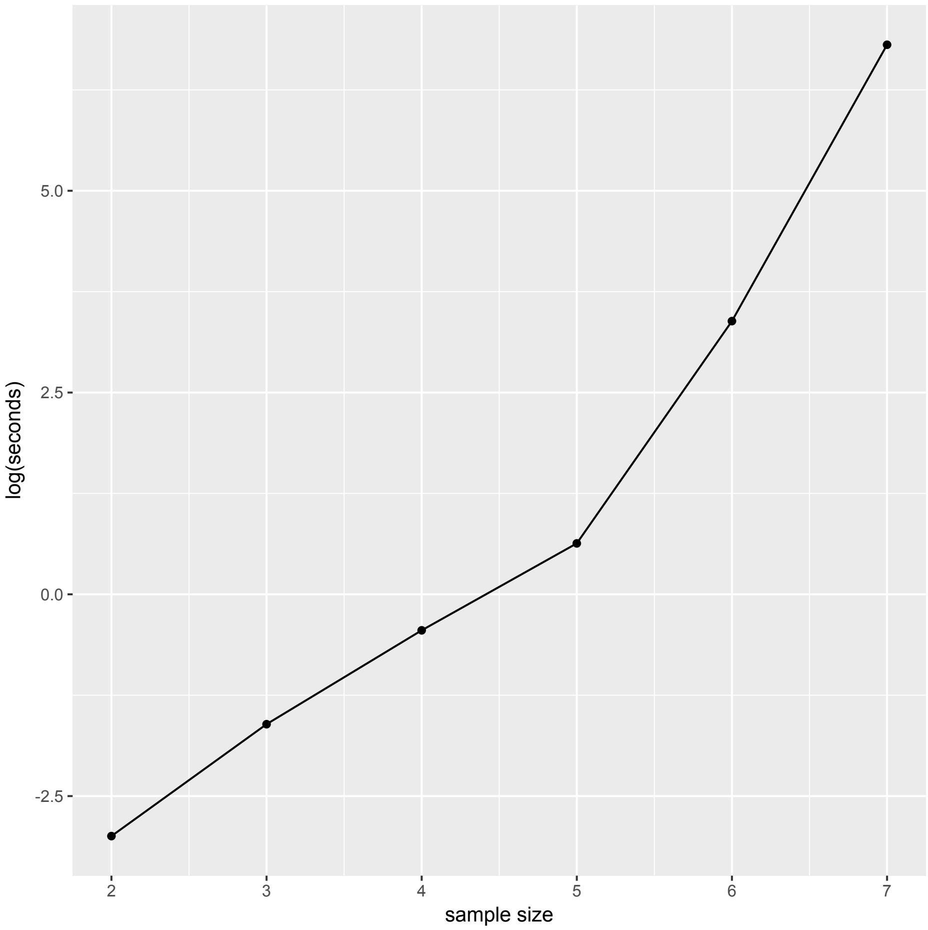

Figure S1: **Calculation time of the AFS as a function of sample size.** Here we show the time it takes to calculate the AFS as sample size for both populations increases equivalently. All jobs were run on 12 cores. The y-axis is shown on a log scale.

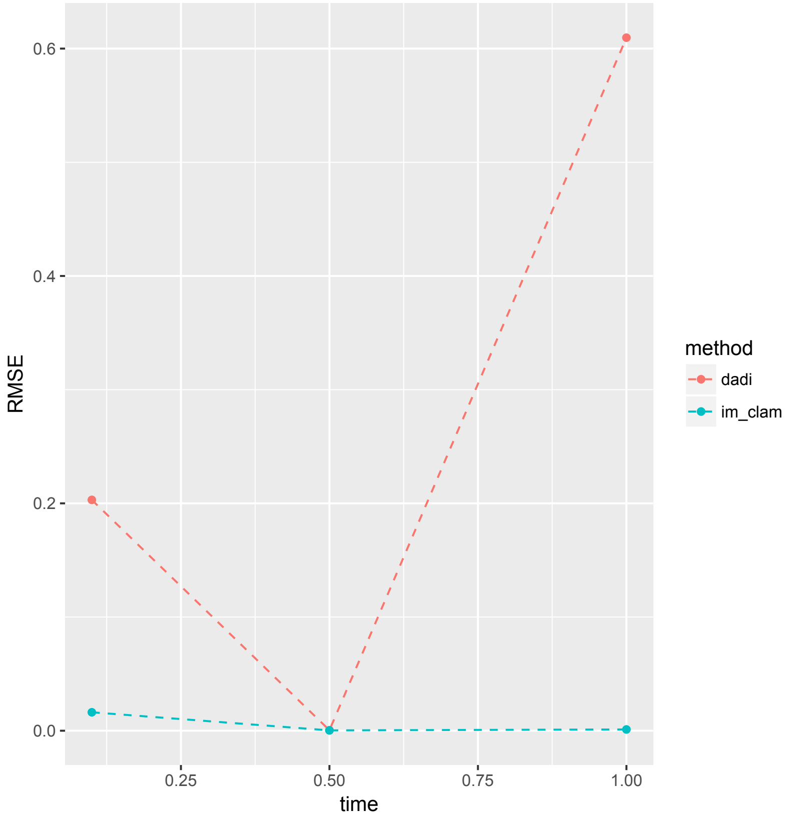

Figure S2: **Accuracy of parameter estimates across divergence time.** Here we show the root mean square error (RMSE) across all parameter estimates as a function of the three parameter sets used in Figure 6. Migration rates, $m_{12} = 1.0; m_{21} = 1.0$, and divergence time varies from left to right $t_{div} = 0.1, 0.5, 1.0$.

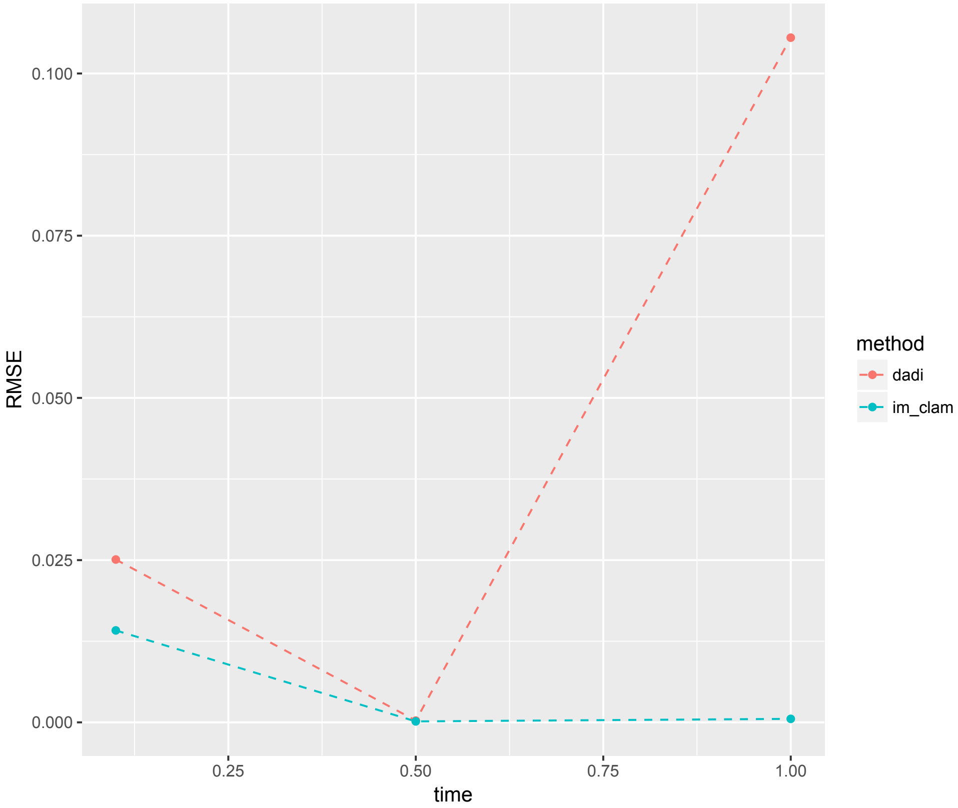

Figure S3: **Accuracy of parameter estimates across divergence time.** Here we show the root mean square error (RMSE) across all parameter estimates as a function of the three parameter sets used in Figure 7. Migration rates, $m_{12} = 1.0; m_{21} = 0.1$, and divergence time varies from left to right $t_{div} = 0.1, 0.5, 1.0$.

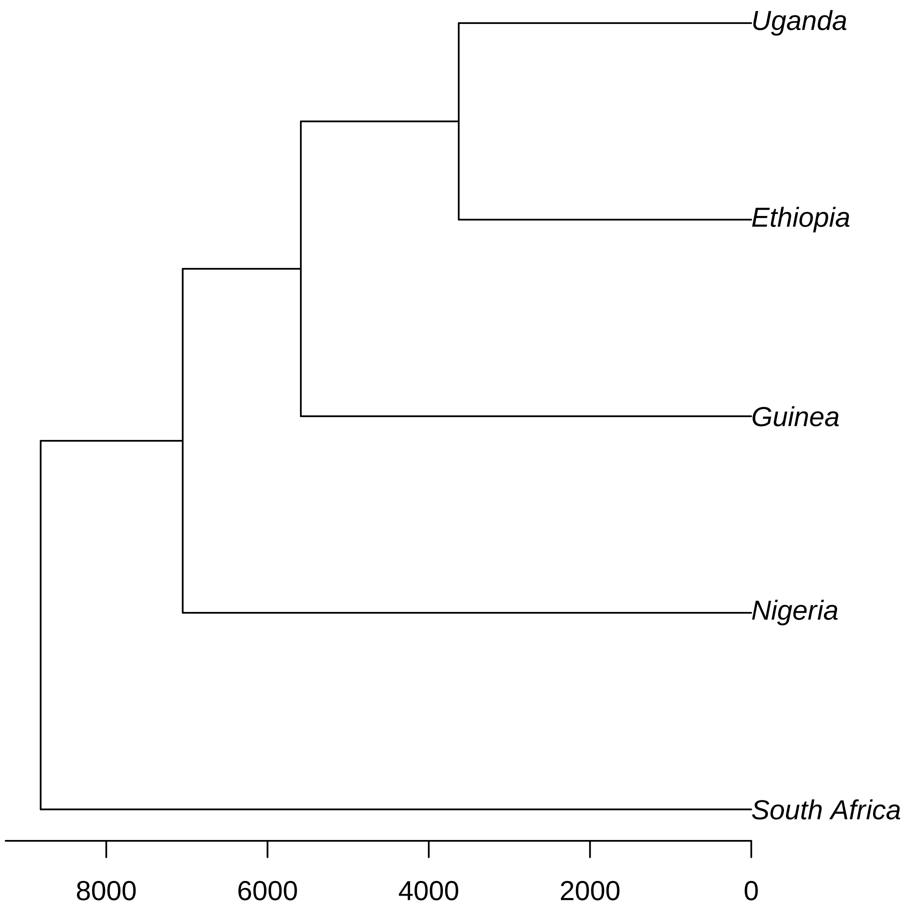

Figure S4: **Distance tree of population divergence times.** Using estimated divergence time in pairwise comparisons as a distance metric, we constructed a tree using UPGMA clustering. Times are in years, as depicted on the x-axis, assuming 15 generations per year, and $u = 5.49 \times 10^{-9}$.
